# Long-Term Patient Satisfaction and Quality of Life Following Breast Reconstruction Using the BREAST-Q: A Prospective Cohort Study

**DOI:** 10.3389/fonc.2022.815498

**Published:** 2022-05-23

**Authors:** Makoto Shiraishi, Yoshihiro Sowa, Itaru Tsuge, Takuya Kodama, Naoki Inafuku, Naoki Morimoto

**Affiliations:** ^1^Department of Plastic and Reconstructive Surgery, University Hospital Kyoto Prefectural University of Medicine, Kyoto, Japan; ^2^Department of Plastic and Reconstructive Surgery, Graduate School of Medicine, Mie University, Mie, Japan; ^3^Department of Plastic and Reconstructive Surgery, Graduate School of Medicine, Kyoto University, Kyoto, Japan; ^4^Department of Plastic and Reconstructive Surgery, Fukuchiyama City Hospital, Fukuchiyama, Japan

**Keywords:** breast reconstruction, tissue expander, breast implant, DIEP, BREAST-Q, health-related quality of life

## Abstract

**Background:**

Breast reconstruction is a promising surgical technique to improve health-related quality of life (HRQoL) in patients with breast cancer. However, the long-term risk factors associated with HRQoL after breast surgery are still unclear. Our aim was to evaluate breast satisfaction and HRQoL following breast reconstruction to identify clinical factors associated with each domain of BREAST-Q in the long-term.

**Methods:**

Patient-reported BREAST-Q outcomes were analyzed 1 and 5 years after breast reconstruction in a single-blinded, prospective study. Multiple regression analysis was performed to identify the risk and protective factors associated with BREAST-Q scores. These scores at 1 and 5 years were also compared across three types of operation: mastectomy only, tissue expander/implant (TE/Imp), and a deep inferior epigastric perforator (DIEP) flap.

**Results:**

Surveys were completed by 141 subjects after 1 year and 131 subjects after 5 years. Compared to mastectomy only, breast reconstruction was significantly associated with greater “Satisfaction with breasts” (TE/Imp, p < 0.001; DIEP, p < 0.001) and “Psychosocial well-being” (TE/Imp, p < 0.001; DIEP, p < 0.001), higher body mass index (BMI) resulted in lower “Satisfaction with breasts” (p = 0.004), and a history of psychiatric or neurological medication was significantly associated with “Physical well-being” at 1-year postoperatively (p = 0.02). At 5 years, reconstructive procedures were significantly positively associated with greater “Satisfaction with breasts” (TE/Imp, p < 0.001; DIEP, p < 0.001) and “Psychosocial well-being” (TE/Imp, p = 0.03; DIEP, p < 0.001), and a bilateral procedure was a significant risk factor for lower “Psychosocial well-being” (p = 0.02).

**Conclusions:**

The results of this study show that breast reconstruction improves “Satisfaction with Breasts” and “Psychosocial well-being” compared to mastectomy. Among all three types of operation, DIEP gave the best scores at 5 years postoperatively. Thus, autologous reconstruction is recommended for promotion of long-term HRQoL after breast surgery.

## Introduction

More than 2 million women worldwide receive a new diagnosis of breast cancer every year (1–3). The number of women surviving breast cancer has increased due to improvements of treatment in many countries, including in Japan (4, 5). Postoperative complications such as lymphoedema, axillary web syndrome (AWS), and fatigue may reduce health-related quality of life (HRQoL) (6–8), but patients also have the opportunity to receive breast reconstruction after mastectomy, which can significantly improve HRQoL (9). Factors associated with HRQoL include satisfaction with appearance, psychological well-being, and physical function. In patients with breast cancer, some studies have shown that aesthetic outcome also influences HRQoL (10, 11).

Multiple questionnaires have been used to measure patient-reported outcomes (PROs) after breast surgery for patients with breast cancer. However, until the turn of the century, few instruments had sufficient evidence for specific use in these patients due to limitations in certain areas, including aesthetics and body perception (12). In 2009, the BREAST-Q questionnaire was developed to meet this need, as a validated PRO measurement specific to breast surgery. Since its release, the BREAST-Q has greatly improved studies of satisfaction with breast surgery from the patient’s perspective (13–16).

Previous studies using the BREAST-Q questionnaire have established that breast reconstruction provides higher levels of patient satisfaction. Most of these studies had short follow-up periods of up to 1 year and limited comparison groups (17–25). In addition, satisfaction with breast reconstruction may change, even over a short period of time (26–28). Long-term satisfaction is important after breast reconstruction, but how satisfaction and HRQoL change years after the initial operation is still unclear.

To investigate these issues further, we performed a long-term prospective survey of patients with breast cancer who underwent breast surgery including breast reconstruction. The objective was to evaluate HRQoL in a Japanese population following breast reconstruction to identify clinical factors that predict higher or lower BREAST-Q scores in long-term survivors.

## Material and Methods

### Subjects and Experimental Design

We prospectively analyzed clinical data for all consecutive patients with breast cancer who underwent breast reconstruction performed by three surgeons at a single center from January 2016 to April 2017. Patients were enrolled in the study if they fulfilled the following criteria: (1) age ≥18 years, (2) undergoing mastectomy only or first-time unilateral or bilateral post-mastectomy breast reconstruction using a tissue expander/implant (TE/Imp) or a deep inferior epigastric perforator (DIEP) flap, and (3) not meeting exclusion criteria of surgical complications such as implant loss or flap loss that could affect long-term results, death, or a poor understanding of the study due to severe neurological or psychiatric disorders. For power analysis, a 10-point difference in HRQoL (BREAST-Q) score was taken to indicate a clinically relevant difference (minimally important difference: MID) based on a previous study (29). Using alpha of 0.05, a standard deviation of 5-10 points from our previous study (30) and beta of 0.80, at least 34 patients per arm were required for significance. Advice on statistical analysis was provided by Statista (Kyoto, Japan), a medical statistics support company. As the scheduled date of closure was reached, enrollment was stopped in April 2017 before reaching the planned sample size.

### Data Collection and Measurements

All subjects provided demographic data. Smoking was divided into past and current. Body mass index (BMI) was calculated as weight in kilograms divided by height in meters squared. Clinical characteristics, type of breast surgery, therapy after mastectomy (radiotherapy, chemotherapy, and/or hormone therapy), and history of psychiatric or neurological illness and medication were obtained from medical records.

### BREAST-Q Survey

The BREAST-Q is a validated PRO measure developed at Memorial Sloan Kettering Cancer Center and the University of British Columbia (13, 14). We focused on three BREAST-Q domains: “Satisfaction with breasts”, “Psychosocial well-being”, and “Physical well-being”. Each domain score was obtained by transforming the scale item responses with the Q-score software program. The transformed scores range from 0 to 100 and higher scores indicated greater satisfaction or QOL. The Japanese version of the BREAST-Q survey was administered prior to surgery after consultation with the surgical oncologist and plastic surgeon, and at 1 and 5 years after completion of surgery (31). At these time points, surveys were given to patients at an office visit or mailed to the patient’s home.

### Statistical Analysis

Statistical analyses were performed using JMP Pro v.14.0 (SAS Institute Inc., Cary, NC) and SPSS v.26.0 (IBM Corp., Armonk, NY). Continuous variables are shown as the mean ± standard deviation (SD) and categorical variables as a number (percentage). A multiple linear regression model was constructed for identification of significant factors for HRQoL. A Mann-Whitney U test was used to compare data between years, and a *post-hoc* Tukey test was used for comparison between operative procedures. P < 0.05 was considered to be significant in all analyses.

### Ethics Approval

All procedures were approved by the local research ethics committee (Kyoto Prefectural University of Medicine: IRBMED Number ERB-C-563-1) and were conducted in accordance with the Declaration of Helsinki. Informed consent was obtained from all subjects.

## Results

**Figure 1:**
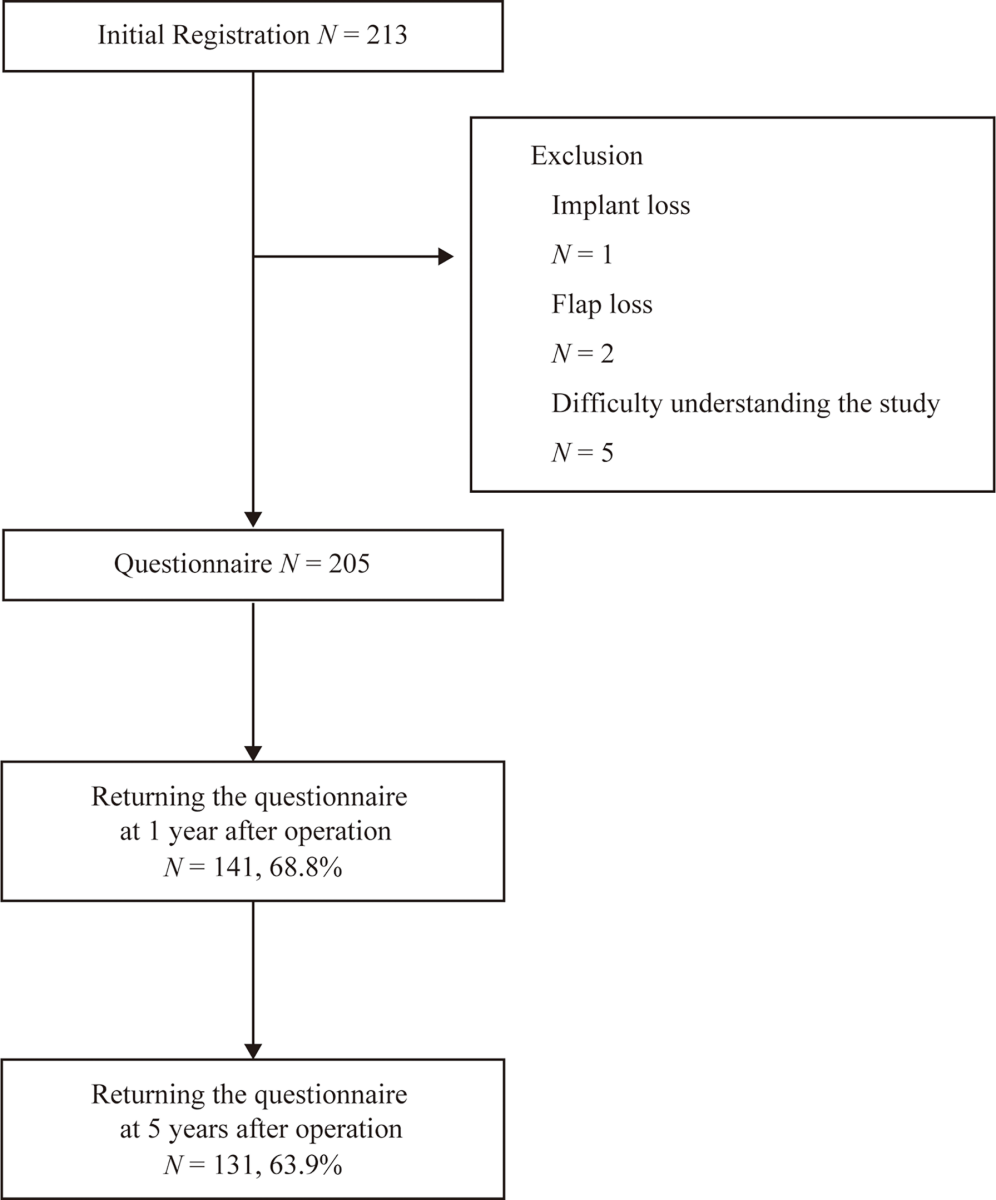
Inclusion and exclusion of subjects.

Among 213 potential subjects, 8 were excluded due to implant loss (n=1), flap loss (n=2), and difficulty understanding the study because of severe neurological or psychiatric disorders (n=5). All patients received immediate reconstruction. Questionnaire surveys were sent to the home addresses of 205 subjects in the year after the operation. Written informed consent and answers were obtained from 141 at 1 year and 131 at 5 years postoperatively, giving response rates of 68.8% and 63.9%, respectively (**Figure 1**).

The demographic and clinical characteristics of the 141 subjects at 1 year and 131 subjects at 5 years are shown in **Table 1**. The subjects were 53.0 ± 12.9 years old and had a BMI of 22.3 ± 3.41 kg/m^2^. The surgical procedures were TE/implant reconstruction (27.4%), mastectomy only (35.0%), and DIEP flap reconstruction (37.6%). Most patients underwent unilateral surgery (94.9%).

**Table 1:** Demographics of the subjects.

Item	Responders after 1 year		Responders after 5 years	
	N	Percent	N	Percent
Number of patients	141	100%	131	100%
Age (years), mean (SD)	53.2 (13.1)		52.8 (12.8)	
BMI (kg/m^2^), mean (SD)	22.0 (3.41)		22.2 (3.45)	
Lesion				
Bilateral	8	5.7%	7	5.3%
Unilateral	133	94.3%	124	94.7%
Type of operation				
Mastectomy only	51	36.2%	45	34.4%
TE/Imp	56	39.7%	53	40.5%
DIEP	34	24.1%	33	25.2%
Radiotherapy				
No	108	76.6%	99	75.6%
Yes	33	23.4%	32	24.4%
Chemotherapy/hormone therapy				
No	78	55.3%	68	51.9%
Yes	63	44.7%	63	48.1%
Smoking				
Never	108	76.6%	100	76.3%
Past	30	21.3%	29	22.1%
Current	3	2.1%	2	1.5%
Psychotic/neurological medical history or medication				
No	133	94.3%	123	93.9%
Yes	8	5.7%	8	6.1%

SD, Standard deviation; BMI, Body mass index; TE/Imp, tissue expander/implant; DIEP, deep inferior epigastric perforator.

Regression analyses for patient-reported aesthetic satisfaction across 3 domains (“Satisfaction with breasts”, “Psychosocial well-being”, and “Physical well-being”) with mastectomy only, TE/Imp and DIEP at 1- and 5-year follow-up after surgery are listed in **Table 2**. These data were controlled for age, BMI, laterality, type of operation, radiation, chemotherapy, smoking, and psychotic/neurological medical history or medication.

**Table 2:** Multiple regression analysis of factors associated with increased satisfaction or QOL for BREAST-Q domains.

Independent Variable	Satisfaction with breasts				Psychological well-being				Physical well-being			
	Year 1		Year 5		Year 1		Year 5		Year 1		Year 5	
	Coefficient	P value	Coefficient	P value	Coefficient	P value	Coefficient	P value	Coefficient	P value	Coefficient	P value
Age	0.03	0.73	0.12	0.23	0.06	0.55	-0.07	0.51	-0.12	0.24	-0.78	0.49
BMI	-0.20	0.004^**^	-0.07	0.40	-0.07	0.39	-0.02	0.85	0.01	0.91	0.17	0.08
Lesion												
Unilateral	Reference		Reference		Reference		Reference		Reference		Reference	
Bilateral	-0.06	0.35	-0.10	0.25	-0.12	0.15	-0.11	0.15	0.00	0.99	-0.07	0.53
Type of Operation												
Mastectomy Only	Reference		Reference		Reference		Reference		Reference		Reference	
TE/ Imp	0.61	< 0.001^***^	0.46	< 0.001^***^	0.43	< 0.001^***^	0.25	0.03^*^	0.02	0.88	-0.08	0.53
DIEP	0.64	< 0.001^***^	0.64	< 0.001^***^	0.35	< 0.001^***^	0.40	< 0.001^***^	0.17	0.11	-0.09	0.43
Preoperative Radiation												
No	Reference		Reference		Reference		Reference		Reference		Reference	
Yes	0.02	0.79	0.10	0.26	0.01	0.87	0.12	0.17	0.08	0.37	0.16	0.10
Chemotherapy												
No	Reference		Reference		Reference		Reference		Reference		Reference	
Yes	0.05	0.50	0.12	0.14	0.17	0.06	-0.05	0.60	-0.05	0.60	0.07	0.44
Smoking												
Never	Reference		Reference		Reference		Reference		Reference		Reference	
Past	0.94	0.17	-0.08	0.29	0.10	0.23	0.11	0.18	-0.07	0.46	-0.04	0.63
Current	-0.54	0.43	0.03	0.71	0.06	0.47	0.05	0.57	-0.07	0.54	-0.01	0.93
Psychotic/Neurological Medical History or Medication												
No	Reference		Reference		Reference		Reference		Reference		Reference	
Yes	-0.02	0.83	0.03	0.73	-0.05	0.56	-0.15	0.84	0.20	0.02^*^	0.17	0.08

BMI, Body mass index; TE/Imp, tissue expander/implant; DIEP, deep inferior epigastric perforator.

^*^P < 0.05. ^**^P < 0.005. ^***^P < 0.001.

At 1-year postoperatively, “Satisfaction with breasts” was significantly impaired in patients with higher BMI (coefficient (β) -0.20, 95% confidence interval (CI) -1.89 to -0.38, p = 0.004). Compared to mastectomy only, TE/Imp and DIEP were both positively associated with “Satisfaction with breasts” (TE/Imp: β -0.61, 95%CI 16.62 to 30.51, p < 0.001; DIEP: β 0.64, 95% CI 21.31 to 35.81, p < 0.001) and “Psychosocial well-being” (TE/Imp: β 0.43, 95% CI 8.71 to 26.80, p < 0.001; DIEP: β 0.35, 95% CI 7.05 to 25.61, p < 0.001) at 1 year. History or medication for a psychotic/neurological condition was associated with greater “Physical well-being” (β 0.20, 95% CI 2.00 to 22.91, p = 0.02) at 1 year. At 5 years, compared to mastectomy only, TE/Imp and DIEP were positively associated with “Satisfaction with breasts” (TE/Imp: β 0.46, 95% CI 7.62 to 21.62, p < 0.001; DIEP: β 0.64, 95% CI 15.82 to 29.52, p < 0.001) and “Psychosocial well-being” (TE/Imp: β 0.25, 95%CI 0.95 to 20.29, p = 0.03; DIEP: β 0.40, 95%CI 9.32 to 28.53, p < 0.001). In addition, “Psychosocial well-being” significantly improved in patients with a bilateral procedure at 5 years (β 0.20, 95% CI -34.31 to -2.90, p = 0.02). No factors were significantly associated with “Physical well-being” at 5 years.

**Figure 2:**
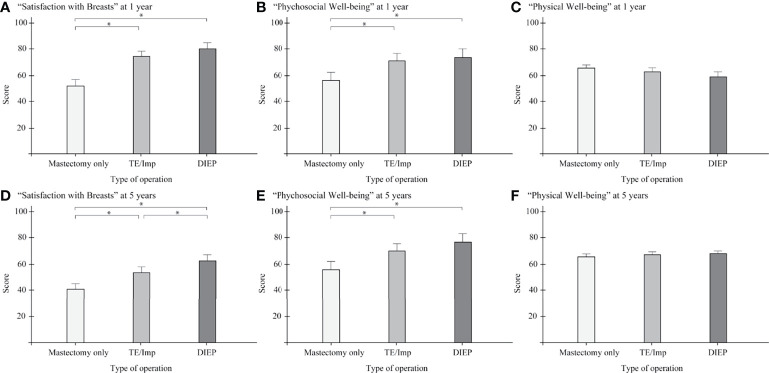
Comparison of BREAST-Q scores among operative procedures in each year, with 95% confidence intervals. **(A)** “Satisfaction with breasts” at 1 year; **(B)** “Psychosocial well-being” at 1 year; **(C)** “Physical well-being” at 1 year; **(D)** “Satisfaction with breasts” at 5 years; **(E)** “Psychosocial well-being” at 5 years; **(F)** “Physical well-being” at 5 years. TE/Imp, tissue expander/implant; DIEP, deep inferior epigastric perforator. *P < 0.05.

Comparisons of BREAST-Q scores among operative procedures in each year are shown in **Figure 2**. Mastectomy scored significantly lower than TE/Imp and DIEP for “Satisfaction with breasts” and “Psychosocial well-being” (both p < 0.001) at 1 year (all p < 0.001; **Figures 2A, B**) and 5 years (all p < 0.001, except p = 0.007 vs. DIEP for “Satisfaction with breasts”; **Figures 2D, E**). In addition, at 5 years, DIEP scored significantly higher than TE/Imp for “Satisfaction with breasts” (p < 0.001) (**Figure 2D**). Detailed results are shown in **Table S1**.

**Figure 3:**
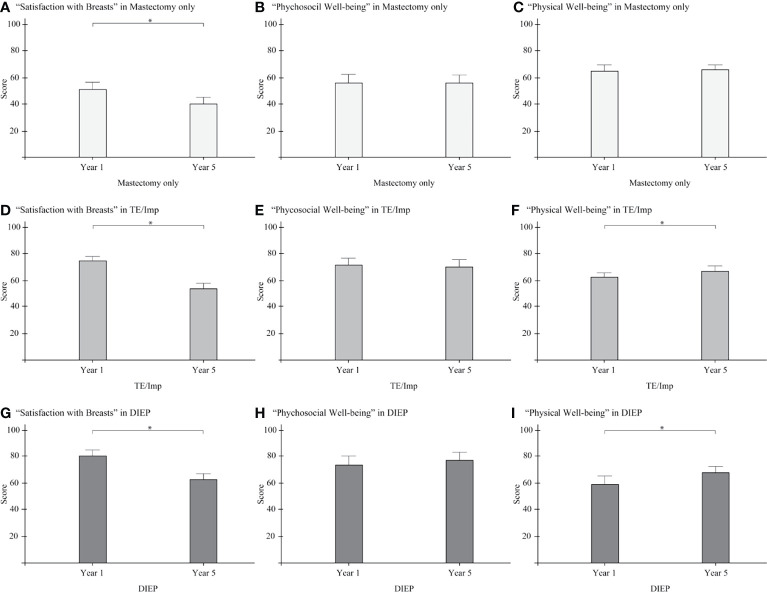
Comparison of BREAST-Q scores in 1- and 5-year follow-up evaluations for three operative procedures, with 95% confidence intervals. **(A)** “Satisfaction with breasts” in mastectomy only; **(B)** “Psychosocial well-being” in mastectomy only; **(C)** “Physical well-being” in mastectomy only; **(D)** “Satisfaction with breasts” in TE/Imp; **(E)** “Psychosocial well-being” in TE/Imp; **(F)** “Physical well-being” in TE/Imp; **(G)** “Satisfaction with breasts” in DIEP; **(H)** “Psychosocial well-being” in DIEP; **(I)** “Physical well-being” in DIEP. TE/Imp, tissue expander/implant; DIEP, deep inferior epigastric perforator. *P < 0.05.

Comparisons of BREAST-Q scores between 1- and 5-year follow-up evaluations for each operative procedure are shown in **Figure 3**. Scores at 5 years were significantly lower than those at 1 year for “Satisfaction with breasts” for all three procedures (mastectomy only, p = 0.012; TE/Imp p < 0.001; DIEP, p < 0.001; **Figures 3A, D, G**) and for “Physical well-being” for two procedures (TE/Imp, p = 0.007; DIEP, p = 0.008; **Figures 3E, H**). Detailed results are shown in **Table S2**.

## Discussion

In this study, general and aesthetic satisfaction with breast operations including reconstruction were investigated at 1 and 5 years postoperatively in Japanese women. The main finding was that the type of operation was significantly associated with HRQoL-related domains of BREAST-Q at both 1 and 5 years in multivariate analysis. Over time, all surgical procedures had lower scores for “Satisfaction with breasts”, but “Psychosocial well-being” was maintained and “Physical well-being” improved after TE/Imp and DIEP. “Satisfaction with breasts” and “Psychosocial well-being” were lowest for mastectomy only at 1 and 5 years, and “Satisfaction with breasts” at 5 years was best after DIEP.

To our knowledge, there have been few long-term studies of HRQoL after breast surgery. Long-term evaluations of 5 years or more that have been performed are shown in **Table 3** (32, 33, 35, 36). In the current study, the response rate and score for “Satisfaction with breasts” declined from postoperative year 1 to year 5. The lower response rate is consistent with previous studies showing a decline in response rate for PROs over time, as patients lose interest in the aesthetic impact of breast surgery (37–41). A lower score at a later time has also been found previously (42, 43) and is due to patients becoming used to the results of reconstruction over time. Thus, the time after the operation is an important factor associated with HRQoL.

**Table 3:** Comparison of long-term breast reconstruction studies using BREAST-Q.

Reference	Patient Base	Number of Patients	BREAST-Q Postop Follow-up Period	Scales	Protective factors	Risk factors
Hu et al. (32)	TE/Imp and TRAM	219	6.5 years^*^	Satisfaction with breasts	TRAM	TE/Imp; early postoperative period
Ledibabari et al. (33)	Mammoplasty	70	6 years^*^	Satisfaction with breasts	None	Obesity
Ticha et al. (34)	Implant-based reconstruction, abdominal-based autologous reconstruction, and combined reconstruction (with implant and LD flap or implant and TDAP flap)	110	5 years	Satisfaction with breasts	Abdominal-based autologous reconstruction	None
				Psychosocial well-being	Abdominal-based autologous reconstruction	None
				Physical well-being	Abdominal-based autologous reconstruction	None
Dominici et al. (35)	Mastectomy, radiotherapy, and autologous flap	290	5.8 years^**^	Satisfaction with Breasts	None	TE/Imp
				Psychosocial well-being	None	None
				Physical well-being	None	Complex reconstruction (vs. autologous)

TE/Imp, tissue expander/implant; DIEP, deep inferior epigastric perforator; TRAM, transverse rectus abdominis myocutaneous; LD, latissimus dorsi flap; TDAP, thoracodorsal artery perforator flap.

^*^Mean; ^**^Median.

Among patient factors, higher BMI was significantly negatively associated with “Satisfaction with breasts” at 1-year postoperatively, but not at 5 years. This is consistent with several reports showing that high BMI is an independent risk factor for lower satisfaction (44–46). However, most of these studies did not obtain baseline data using the preoperative module of BREAST-Q. Previous reports have also shown that obese patients tend to have higher rates of postoperative complications. However, in the current study, higher BMI at 5 years was not a risk factor, which suggests that these complications had resolved or that patients had become used to their postoperative status.

Psychotic/neurological medical history or medication was found to be a significant risk factor that lowers “Physical well-being” at 1 year after breast surgery. The questions in the “Physical well-being” domain are related to physical problems, including pain in the chest, back, abdomen or skin. Among psychological factors, preoperative levels of depression, anxiety, and psychological vulnerability to aberrant pain perception have been reported to be significantly associated with greater postoperative pain intensity, which may decrease physical morbidity (47–51). However, in a previous study, we found no significant association of psychotic/neurological medical history or medication with postoperative pain at one year after breast surgery in a similar cohort of Japanese patients to that in the current study (52). Thus, more psychiatrically oriented pain such as chronic postsurgical pain (CPSP) may have a negative effect on “Physical well-being” of patients (53).

A bilateral procedure was significantly associated with “Psychosocial well-being” at 5 years after breast surgery. Several studies focusing on bilateral breast operations have concluded that “Psychosocial well-being” after surgery significantly improves compared to the preoperative level (54, 55). These findings suggest that patients who underwent bilateral reconstruction were more satisfied due to improved symmetry and a superior aesthetic appearance.

Age was not found to be a significant factor associated with HRQoL among breast cancer survivors in the current study. However, this is still controversial because some reports indicate that implant and autogenous tissue techniques are associated with aging processes that can affect aesthetic appearance (56–60), whereas other studies did not find significant age-related differences using BREAST-Q (61–64). Reconstructive surgeons may avoid autologous reconstruction in older women due to complications following longer anesthetic times, but our results indicate that autologous procedures can be viable choices in older patients, given that age is not associated with greater risks and that autologous reconstruction in this population still achieves high HRQoL.

In this study, we focused on general and aesthetic well-being after breast surgery. Postoperative complications such as lymphedema, AWS, and fatigue can lower HRQoL, but intervention through rehabilitation can improve satisfaction (6–8). Risk factors for each complication have been described (65–68) and there are also several predictive methods for the complications. For example, de Sire et al. found that measuring the upper limb volume using a three-dimensional laser scanner was a reliable way to diagnose breast cancer-related lymphedema (69), and Nevola Teixeira et al. established a self-assessment questionnaire for AWS (70). A combination of BREAST-Q and these diagnostic methods is a promising approach for evaluation of complications.

The main strength of the study is the long-term evaluation of PROs for breast reconstruction. There are several limitations in the study. The main limitation was the response rates of 68.8% at 1 year and 63.9% at 5 years. However, these rates are similar to those in previous reports (71–73). Patients were followed for up to 5 years, but with such a long study period some could not be contacted or may have died, and were lost to follow up. It is also possible that there was a non-response bias, since patients who are still thinking about the complications of their breast reconstruction are more likely to respond to a questionnaire. We also did not examine the baseline status of the patients in terms of their well-being and satisfaction, and we were unable to assess changes in BMI in the postoperative period, which may influence patient satisfaction. However, in a retrospective study, Applebaum et al. found no significant change in BMI over time following implant-based or autologous breast reconstruction (74). The current study was also restricted to a single center with a relatively homogeneous patient population and evaluation, which could lead to potential selection bias. Finally, it was difficult to control for variability in operative techniques of surgeons and management of postoperative complications in statistical analysis.

In conclusion, the BREAST-Q score for “Physical well-being” was maintained at 5 years after breast reconstruction, and breast reconstruction procedures were better than mastectomy for “Satisfaction with breasts” and “Psychosocial well-being”. DIEP had the best scores among the three procedures at 5 years postoperatively. Thus, autologous reconstruction using a DIEP flap is recommended in terms of long-term satisfaction after breast surgery. These results are clinically useful for the choice of operative method by surgeons and patients. However, factors such as ethnic and regional differences may affect the results in other cohorts, and further research is required to promote better satisfaction with HRQoL after breast reconstruction.

## Data Availability Statement

The original contributions presented in the study are included in the article/**Supplementary Material**. Further inquiries can be directed to the corresponding author.

## Ethics Statement

The studies involving human participants were reviewed and approved by the ethics committee of Kyoto Prefectural University of Medicine, IRBMED Number ERB-C-563-1. The patients/participants provided their written informed consent to participate in this study.

## Author Contributions

MS and YS contributed to the conceptualization, methodology, investigation, and revision of the manuscript. MS wrote the original draft. TK and NI contributed to the collection of patient data, the investigation, and revision of the manuscript. IT and NM contributed to the revise of the article.

## Funding

Costs related to statistical analysis and manuscript publication were funded through the department of Plastic and Reconstructive Surgery, Kyoto Prefectural University of Medicine.

## Conflict of Interest

The authors declare that the research was conducted in the absence of any commercial or financial relationships that could be construed as a potential conflict of interest.

## Publisher’s Note

All claims expressed in this article are solely those of the authors and do not necessarily represent those of their affiliated organizations, or those of the publisher, the editors and the reviewers. Any product that may be evaluated in this article, or claim that may be made by its manufacturer, is not guaranteed or endorsed by the publisher.
