# Supplementary material for: Long-Term Patient Satisfaction and Quality of Life Following Breast Reconstruction Using the BREAST-Q: A Prospective Cohort Study
Source: Front Oncol. 2022 May 23;12:815498. doi: 10.3389/fonc.2022.815498 (PMC9178786; doi:10.3389/fonc.2022.815498)
Supplement: Supplementary file 1 [file Table_1.docx]

**Table S1.** Results of ANOVA and post hoc Tukey tests for comparison of BREAST-Q scores among operative procedures in each year

| BREAST-Q domain | Year 1 | | | Year 5 | | |
| --- | --- | --- | --- | --- | --- | --- |
|  | Mean Difference | SE | P value | Mean Difference | SE | P value |
| Satisfaction with breasts | | | |  |  |  |
| DIEP - Mastectomy only | 28.25 | 3.34 | < 0.001^***^ | 21.66 | 3.10 | < 0.001^***^ |
| TE/Imp - Mastectomy only | 22.94 | 2.90 | < 0.001^***^ | 13.12 | 2.79 | < 0.001^***^ |
| DIEP – TE/Imp | 5.31 | 3.25 | 0.236 | 8.54 | 2.99 | 0.014^**^ |
| Psychosocial well-being | | | |  |  |  |
| DIEP - Mastectomy only | 17.08 | 4.26 | < 0.001^***^ | 21.25 | 4.45 | < 0.001^***^ |
| TE/Imp - Mastectomy only | 15.53 | 3.75 | < 0.001^***^ | 14.36 | 3.96 | 0.001^*^ |
| DIEP – TE/Imp | 1.54 | 4.17 | 0.93 | 6.89 | 4.27 | 0.24 |
| Physical well-being | | | |  |  |  |
| DIEP - Mastectomy only | -6.36 | 3.15 | 0.11 | 2.21 | 2.96 | 0.74 |
| TE/Imp - Mastectomy only | -2.62 | 2.76 | 0.61 | 1.81 | 2.64 | 0.77 |
| DIEP - TE/Imp | 3.74 | 3.10 | 0.45 | -0.40 | 2.85 | 0.99 |

SE: Standard error; TE/Imp: tissue expander/implant; DIEP: deep inferior epigastric perforator.

^*^ *P* < .05. ^**^ *P* < .005. ^***^ *P* < .001.
