# Supplementary material for: Long-Term Patient Satisfaction and Quality of Life Following Breast Reconstruction Using the BREAST-Q: A Prospective Cohort Study
Source: Front Oncol. 2022 May 23;12:815498. doi: 10.3389/fonc.2022.815498 (PMC9178786; doi:10.3389/fonc.2022.815498)
Supplement: Supplementary file 2 [file Table_2.docx]

**Table S2.** Results of Mann-Whitney U tests for comparison of BREAST-Q scores in 1- and 5-year follow-up evaluations for three operative procedures

| Procedure | Year 1 | Year 5 | P value |
| --- | --- | --- | --- |
| Mastectomy Only | | | |
| Satisfaction with breasts | 51.91 ± 17.73 | 40.95 ± 14.07 | 0.002^**^ |
| Psychosocial well-being | 56.73 ± 20.54 | 56.14 ± 20.21 | 0.84 |
| Physical well-being | 65.54 ± 14.41 | 65.93 ± 12.62 | 0.99 |
| TE/Imp | | | |
| Satisfaction with breasts | 74.85 ± 12.55 | 54.08 ± 13.00 | < 0.001^***^ |
| Psychosocial well-being | 72.26 ± 17.86 | 70.50 ± 19.79 | 0.78 |
| Physical well-being | 62.92 ± 11.40 | 67.74 ± 13.21 | 0.002^**^ |
| DIEP | | | |
| Satisfaction with breasts | 80.16 ± 13.57 | 62.61 ± 12.84 | < 0.001^***^ |
| Psychosocial well-being | 73.80 ± 18.87 | 77.39 ± 16.77 | 0.48 |
| Physical well-being | 59.18 ± 17.80 | 68.14 ± 12.39 | 0.003^**^ |

TE/Imp: tissue expander/implant; DIEP: deep inferior epigastric perforator.

^*^ *P* < .05. ^**^ *P* < .005. ^***^ *P* < .001.

Values are presented as mean ± standard deviation.
